# Case report for an intervention for scientific pressure by brief strategic withdrawal

**DOI:** 10.3389/fpsyt.2026.1862209

**Published:** 2026-08-26

**Authors:** Lin Meng, Shiqi Wu, Zhen Wu

**Affiliations:** 1 Department of Psychosomatic Medicine, The Second Affiliated Hospital of Tianjin University of Traditional Chinese Medicine, Tianjin, China; 2 Department of Psychology, Tianjin University of Technology and Education, Tianjin, China

**Keywords:** brief strategic withdrawal, intervention, postgraduate, scientific anxiety, scientific pressure, strategic family therapy

## Abstract

**Background and aims:**

With the increasing number of postgraduates being admitted, the scientific and academic pressure faced by this group is becoming more obvious, causing more concerns among people in tertiary education. This study is an exploratory, context-rich single-case report. This research aims at supplement the research deficiency in releasing pressure related to academic pressure. Based on *counter-intuitive* adopted in strategic family therapy, we designed a five-day strategic withdrawal, exploring the effects it caused on mid-term postgraduates in terms of releasing pressure.

**Methods:**

A single-case pretest-posttest design was adopted. A 23-year-old unmarried female master’s student in social science at the middle stage of her research project. She passed standardized screening procedures and satisfied all pre-set inclusion criteria. She was instructed to suspend stress-triggering behaviors such as thesis writing and literature searching. Quantitative data and qualitative materials were collected using the Research Anxiety Scale, the Challenge-Hindrance Research Stressors Scale, and diaries at three time points: before withdrawal (T1), after withdrawal (T2), and two weeks after withdrawal (T3). The withdrawal effects and their sustainability were comprehensively evaluated.

**Results:**

Brief strategic withdrawal intervention is correlated with decreased research anxiety levels supported by quantitative longitudinal data; it yields alleviating effects on hindrance research stress, yet produces no improvement in challenge research stress, and all these claims are backed by corresponding evidenced data.

**Conclusion:**

According to the research, brief strategic withdrawal is an accessible intervention that exerts beneficial effects on mitigating research anxiety and stress among postgraduates,. creating positive reference for developing strategic therapy. This study is limited by its single-participant sample and short follow-up duration, so caution should be exercised when generalizing the findings. Large-sample randomized controlled trials are needed in future research to clarify the universal effectiveness of this intervention.

## Introduction

In recent years, the enrollment scale of graduate students in China has continued to expand. Data show that the number of enrolled postgraduate students increased from 164,100 in 2002 to 1,103,500 in 2022, and the number of current master’s students rose from 392,100 to 3,097,500, representing growth rates of 572.46% and 689.97%, respectively (1). With the continuous expansion of postgraduate enrollment, the research pressure faced by this group has become increasingly prominent and has drawn considerable attention in higher education.

Chronic research pressure not only impairs physical and mental health, leads to research burnout and reduced research performance, but also poses a serious threat to graduate students’ academic development and psychological well-being (2). This progressive deterioration follows a three-stage vicious cycle: persistent research tasks accumulate hindrance stressors, prolonged stress triggers rumination and research anxiety, and the absence of effective psychological recovery further reduces research self-efficacy and amplifies academic distress (3–9).Cavanaugh introduced the concept of dual stress, proposing that the stress experienced by graduate students can be divided into challenge stressors and hindrance stressors based on their sources (10). Challenge stressors include academic workload, time pressure, research speed, project complexity, and research responsibility, which promote personal growth and research motivation. Hindrance stressors include interpersonal conflict, insufficient resources, job insecurity in research, and other factors that lead to reduced engagement in learning or research activities (5, 11, 12). Empirical research has revealed that both challenge stressors and hindrance stressors are significantly positively correlated with emotional exhaustion among Chinese medical graduate students (7). Another study showed that challenge research stressors have a significant positive impact on research performance, whereas hindrance research stressors have a significant negative impact on research performance (13).Although challenge stressors are generally considered *good stressors* with positive effects, while hindrance stressors are considered *bad stressors*, it is worth noting that even good stressors may have negative consequences (14).

Existing intervention approaches for graduate research anxiety fall into three mainstream categories. First, an experiential curriculum integrating body and mind, which combines physical awareness with medical humanities content, effectively reduces anxiety levels (15). Second, when Mindfulness-Based Stress Reduction (16) is used together with Cognitive Behavioral Therapy (CBT), it not only significantly lowers anxiety but also improves academic performance and overall well-being (17). Third, structured physical exercise has a good effect on academic anxiety, with mind-body integrated exercises showing even better outcomes (18, 19). Building on the above, this study designs an withdrawal based on the principles of Brief Strategic Family Therapy (BSFT) (20), namely short duration and change through action. The withdrawal advocates abandoning ineffective traditional methods and adopting a counter-intuitive logic, turning resistance into an advantage (21). Specifically, it introduces planned, brief, and structured deliberate pauses in specific research activities (e.g., thesis writing, literature searching) to interrupt rigid behavioral patterns under continuous stress. This approach is termed *Brief Strategic Withdrawal*. Such an withdrawal is problem-focused and does not emphasize long-term working through the root causes of stress. Instead, it designs creative, non-traditional, even paradoxical tasks to break dysfunctional behavioral sequences. Through active behavioral withdrawal, it rebuilds the individual’s sense of control over the pace of research, thereby alleviating research anxiety in a relatively short time and creating conditions for more systematic stress management later.

Previous researches on strategic family therapy has mostly focused on complex psychological disorders (e.g., anorexia nervosa, schizophrenia, substance abuse) and has often been presented in the form of case reports with insufficient methodological rigor. This study innovatively applies the brief strategic withdrawal to research pressure management to alleviate research anxiety, thereby enriching the application scenarios of strategic family therapy.

## Case report

The research subject was a 23-year-old unmarried female master’s student majoring in social science who was in the middle stage of her thesis research project. To systematically gather the participant’s mental health background as baseline data, a trained researcher carried out an individual semi-structured interview one day prior to the five-day formal strategic withdrawal intervention. The interview protocol covered three core dimensions: prior persistent anxiety episodes, long-term emotional distress induced by academic stress, and any previous mental adjustment attempts or formal psychological intervention experiences. After the interview, the participant completed standardized screening assessments and met all pre-established inclusion criteria. She reported no history of severe mental disorders; at baseline, she only presented symptoms associated with research stress and showed no signs of chronic psychological pathology. To ensure anonymity, personal identifiers such as name and university are not disclosed.

Prior to the intervention, the participant suffered from chronic distress stemming from thesis-related pressures. Pre-intervention diary entries documented her baseline state across three domains: overlapping internship, dissertation and competition tasks resulted in continuous cognitive depletion and emotional exhaustion; negative cognition was dominated by automatic catastrophic thinking and repetitive academic rumination; anxiety impaired her ability to concentrate, accompanied by obvious task avoidance, procrastination and low research productivity. These baseline characteristics were used as a benchmark to distinguish genuine psychological and behavioral shifts following the intervention.

This study adopted a single-case pretest-posttest design, with measurements conducted at three time points: one day before the withdrawal (T1), the day after the five-day withdrawal (T2) and on the 14th day after the withdrawal (T3). All measurements were uniformly taken at 8:00 a.m. to control for potential interference from diurnal mood fluctuations on the measurement results.

This withdrawal strictly follows the counter-intuitive logic of strategic family therapy. Targeting the core problem of the participant—behavioral overload leading to anxiety and internal attrition—it establishes an withdrawal framework of behavioral constraint with cognitive retention. On this basis, the protocol clearly defines the boundaries of prohibited and permitted actions. Clear boundaries of permitted and prohibited behaviors were defined throughout the 5-day strategic disengagement period: all hands-on research activities including thesis writing, data analysis, literature retrieval and questionnaire distribution were strictly forbidden, and detailed records of daily activities (leisure, social interaction, physical exercise, etc.) were documented. This approach both breaks the ineffective behavioral cycle and maintains cognitive connection with research tasks. To ensure implementation of the withdrawal, the researcher checks the participant’s compliance daily. The study protocol was approved by the ethics committee before commencement. Prior to the withdrawal, the researcher and the participant sign a *Strategic Withdrawal Experimental Agreement*, which clarifies the rights and obligations of both parties and ensures the participant’s informed consent and the right to withdraw at any time.

This study employed the Research Anxiety Scale developed by Wang Xianya et al. (22), which consists of four items and has a unidimensional structure. The scale uses a Likert 5-point scoring system, with higher scores indicating greater levels of research anxiety. This scale has shown good reliability and validity in previous research (13, 23). Because this research only has one participant, no further reliability testing was conducted, which primarily focused on the analysis of intra-individual longitudinal changes.

The Challenge–Hindrance Research Stressors Scale adopted in this study was developed by Chinese scholar Liu Cong (24). This scale contains a total of 16 items divided into two dimensions, with 8 items for challenge stressors and another 8 items for hindrance stressors. A 5-point Likert scoring system is applied, where higher scores indicate greater perceived research stress, and the scale has satisfactory reliability and validity.

## Research results

### Quantitative results

The withdrawal effect was evaluated by calculating the magnitude and rate of score decline across stages. The specific results are shown in **Table 1**.

**Table 1:** Scale scores and their reduction rates.

Scale type	Pre-Intervention (T1)	Post-Intervention (T2)	week follow-up (T3)	T1–T2 Score Reduction	T1–T2 RCI	T2–T3 Score Reduction	T2–T3 RCI	T1-T3Score Reduction	T1-T3RCI
RAS	19	12	8	7	-4.01	4	-2.29	11	-6.30
TRS	49	41	36	8	-1.97	5	-1.23	13	-3.20
CRS	23	21	19	2	-0.77	2	-0.77	4	-1.54
HRS	26	20	17	6	-2.08	3	-1.04	9	-3.12

RAS, Research Anxiety Scale; TRS, Total Research Stress Scale; CRS, Challenge Research Stress Scale; HRS, Hindrance Research Stress Scale.

Reliable improvement was defined as RCI < −1.96, reliable deterioration as RCI > 1.96, and non-reliable change as −1.96 ≤ RCI ≤ 1.96 (95% confidence level(20).

Reliable Change Index (RCI) analysis (25) demonstrated reliable improvements in research anxiety across T1→T2 (RCI = −4.01), T2→T3 (RCI = −2.29), and the full baseline-to-follow-up span T1→T3 (RCI = −6.30). These results suggest that Brief Strategic Withdrawal stably and persistently mitigates research anxiety, with therapeutic gains and no evidence of symptom rebound.

Marked heterogeneity was found between the two subdimensions of research stress. Hindrance research stress yielded reliable improvement from pre- to post-intervention (T1→T2, RCI = −2.08), with additional mild reductions during follow-up that did not meet the reliable change threshold (T2→T3, RCI = −1.04); the overall shift from baseline to two-week follow-up also represented a reliable improvement (T1→T3, RCI = −3.12). Conversely, challenge research stress displayed non-reliable shifts over both adjacent measurement windows (T1→T2, RCI = −0.77; T2→T3, RCI = −0.77), and the aggregate T1–T3 change still did not satisfy the reliable change criterion (RCI = −1.54).For the overall Research Stress Scale total score, the reduction from baseline to post-intervention nearly hit the reliable change cutoff (T1→T2, RCI = −1.97), followed by modest additional stress relief at follow-up (T2→T3, RCI = −1.23). The cumulative T1-to-T3 change across the full observation period reached the standard for reliable improvement (RCI = −3.20). Overall, the stress-mitigating effect of Brief Strategic Withdrawal is concentrated on hindrance research stress, while the intervention produces no statistically meaningful reliable alterations in challenge research stress.

### Qualitative results

During the 5-day withdrawal, the participant kept a diary documenting subjective experiences, emotional changes, behavioral reactions, and cognitive fluctuations related to research tasks. This study employed qualitative case study (QCS) and phenomenological research (PR) methods to analyze the diary content, with the results serving as a supplement to the quantitative data. QCS treats the complete 5-day intervention as a single continuous case. It provides a holistic temporal framework for analysis and integrates fragmented daily experiences into a coherent developmental trajectory of the case. PR focuses on understanding how individual participants construct subjective meanings of research anxiety and strategic disengagement within the intervention context, exploring internal experiences such as emotional fluctuations and cognitive perceptions embedded in diary texts. Rooted in constructivist epistemology, both approaches value participants’ lived experiences and enable an analysis of dynamically evolving psychological processes throughout the intervention.

Coding was conducted at the level of independent meaning units extracted from diaries. we adopted detailed procedures of inter-coder double coding to implement investigator triangulation and mitigate researcher bias. Specifically, two researchers independently coded all diary data. Subsequently, their coding outcomes were compared; inconsistencies were resolved by revisiting the original diaries and engaging in thorough discussions to reach a unified coding scheme, based on which primary, secondary and higher-order themes were sequentially distilled. The detailed coding procedures are outlined as follows. First, all original diary entries were read repeatedly to achieve immersive familiarity with the full corpus. Second, open coding was performed to extract all meaningful *in-vivo* descriptions from the text, such as “When I turned to fetch a book while studying, I accidentally saw data from my graduation thesis, and anxiety washed over me again. “Third, open codes were categorized and integrated to develop primary themes, for instance, “Interest and curiosity toward scheduled arrangements”. Fourth, primary themes were aggregated into secondary themes according to their internal logical connections and temporal sequence, an example being “Relief and cognitive shifts in the early stage of intervention”. Ultimately, the higher-order theme — “Process and reflections on coping with thesis anxiety via strategic disengagement interventions” — was formulated.

The specific results are shown in **Table 2**.

**Table 2:** Thematic analysis based on daily diaries.

Theme classification Original material
Higher-order theme 1: The process and reflection of coping with thesis anxiety through the “strategic withdrawal” withdrawal.
Sub-theme 1: Relief and cognitive transformation in the early stage of the withdrawal
Primary theme: Interest and curiosity about the agreed plan Original material: "I immediately became interested and gladly agreed. On the one hand, I looked forward to the changes in my mental journey during the agreed period; on the other hand, I was curious about how this agreement could change my anxiety state when facing research."
Primary theme: guilt-free relaxation Original material: “Finally, I don’t have to think about this damn paper! And it’s completely guilt-free.”
Primary theme: improvement in mental and physical state Original material: "That afternoon, I took an exceptionally sound nap — something that was difficult to achieve during peak periods of research stress."
Sub-theme 2: Inner conflicts and emotional fluctuations during the withdrawal period
Primary theme: Accidentally induced anxiety Original material: "While studying, I turned my head to grab a book and inadvertently caught sight of my thesis data. The feeling of anxiety came rushing back again."
Primary theme: Emotional self-isolation Original material: "Just as the anxiety was about to spiral out of control, I told myself: 'Stop thinking about it! I don't have to worry about the paper these days — even if there's a group meeting next time, I don't need to have made any progress.' (I found an excuse to isolate my emotions.)"
Primary theme: Be fully engaged in the moment Original material: “I didn’t think about my thesis at all while hanging out with my friend — I was totally present in our chat.”
Primary theme: Anxiety resurgence at night Original material: "Before going to bed at night, thoughts related to the thesis involuntarily surfaced again... The thesis triggered a stream of random, anxious thoughts."
Sub-theme 3: What the participant did and learned after the withdrawal ended
Primary theme: Take action Original material: "Internship began in the new week, and the test process on me finally ended. That afternoon, I immediately posted to collect data for my thesis on Douban (an online platform for study)..."
Primary theme: Insight through adversity Original material: She said, “Only by actually getting down to work will you no longer fear your thesis; in fact, it is not as difficult as you imagine.” … No matter how many withdrawal methods are applied, none is as effective as confronting the difficulty head-on.
Sub-theme 4: withdrawal effect internalization and strategy formation
Primary Theme: Writing for catharsis and clarification Original Material: “I simply sat up in bed, opened my laptop in the dead of night, and transformed, word by word, the anxiety I had been experiencing during the thesis writing process. After finishing writing, my anxiety was noticeably relieved…”
Primary Theme: Formulating coping strategies (the 5% Principle & the 10-Minute Principle) Original Material: “At the same time, I also figured out two small methods to cope with thesis anxiety and procrastination: one is the 5% principle… the other is the 10-minute principle…”
Primary Theme: Breaking the cycle of internal friction: A Meta cognitive Approach Original Material: “This strategic withdrawal also gave me profound insight: when you hesitate about doing something, there’s no need to act immediately—first think it through… Once you have clarified your thinking, then start decisively. It helped me break the cycle of internal conflict caused by ‘overthinking and underdoing.”

Information about the participant’s daily activities across the whole observation period was extracted from daily reflection diaries. The participant’s regular leisure arrangements included weekly swimming and outdoor socializing with peers. Throughout the study, the participant did not receive formal psychological counselling or targeted professional social support, which rules out external supportive resources as a confounding variable affecting changes in mental wellbeing.

The diary captures the complete psychological journey from anxiety to relief, to relapse, to action, and finally to internalization. The agreement on strategic withdrawal not only alleviated anxiety at the time, but more importantly, served as a behavioral experiment that allowed the individual to experience firsthand the heightened efficiency that comes from separating thinking from action. The idea that “you won’t be afraid once you start doing it” played a key role. The effects of the withdrawal were not limited to progress on the thesis; they also enhanced the individual’s metacognitive abilities (awareness of their own thought and emotional patterns).After the 5-day brief strategic withdrawal, the participant consistently maintained the self-regulation strategies developed during the intervention. The core adaptive pattern of separating academic ideation from mandatory hands-on research tasks including thesis writing and data collection was sustained, and the participant no longer forced themselves to push research progress relentlessly. The participant retained a set of healthy adaptive behaviors, such as scheduling fixed weekly activities including swimming and outdoor socializing with peers. This practice completely eliminated the internalized guilt of regarding rest as academic laziness and established a regular work-rest balance routine.

## Discussion

The thesis-writing phase of the master’s program represents a high-risk period for postgraduate psychological distress. The accumulation of multiple academic and internship commitments continuously depletes cognitive resources, triggering research anxiety and differentiated academic stress (3, 4).

This study constructs a 5-day structured withdrawal protocol of “behavioral restriction with retained cognition” based on the paradoxical intervention theory of Brief Strategic Family Therapy, which bears distinctive strengths (26). First, adhering to the core paradox logic of “taking the opposite approach”, the protocol actively restricts all hands-on research operations to break the self-reinforcing cycle sustained by constant struggle against anxiety. Second, it differs from traditional full psychological withdrawal, which requires complete disengagement from all research-related thinking and conflicts with the prevalent domestic cultural perception that “rest equals academic laziness”, thereby inducing guilt and reducing intervention adherence (6). This protocol permits internal deliberation on research ideas and maintains cognitive connections with research tasks. Such a “legitimate pause” dismantles postgraduates’ misconception that consistent research equals diligence and offers a safe outlet for emotional release. It concretizes the paradoxical logic of strategic family therapy in research stress intervention: instead of actively resolving academic problems, independent emotional regulation is achieved through behavioral adjustments. Third, a continuous 5-day withdrawal forms a stable cycle for cognitive resource restoration, unlike fragmented short breaks. The 14-day follow-up data of this study confirm sustained anxiety relief without rebound, addressing the deficiency of poor long-term efficacy in brief rest interventions. Qualitative diary records show that the participant released emotional tension through socializing, swimming and outdoor recreation during withdrawal, while spontaneously organizing plans for data collection and thesis writing, yielding dual gains of emotional recovery and academic cognitive accumulation — a dual-function pathway rarely explored by existing intervention models.

Longitudinal tracking based on the Reliable Change Index (RCI) across three time points (T1/T2/T3) demonstrates that brief strategic withdrawal yields sustained, long-term alleviation of overall research anxiety. Only transient improvements are observed for hindrance research stress, which dissipate at the two-week follow-up, whereas no statistically reliable changes in challenge research stress are detected throughout all measurement phases. Research anxiety arises from repetitive rumination triggered by compulsive continuous research engagement. By restricting hands-on research activities via paradoxical intervention, this study disrupts the self-reinforcing anxiety loop and facilitates persistent emotional relief. The intervention effect persisted through the two-week follow-up period, and the underlying mechanism may lie in the participant’s consistent adoption of self-help emotional regulation strategies acquired during the intervention phase after the withdrawal ended. The Conservation of Resources Theory (8) provides a framework to interpret these differentiated intervention effects. Hindrance stress stems from objective structural constraints such as ambiguous task allocation within research groups, insufficient academic resources, and prolonged peer review cycles. Brief strategic withdrawal can temporarily ease emotional exhaustion yet cannot eliminate the root stressors. On the contrary, challenge stress originates from intrinsically rewarding academic demands, including thesis innovation targets, publication requirements and independent research exploration, which represent opportunities for resource gain. Although the intervention interrupts maladaptive emotional rumination, it does not remove the core academic goals and developmental demands that constitute challenge stress. Accordingly, the intervention does not reduce challenge research stress. Importantly, this outcome carries positive implications: moderate challenge stress helps maintain intrinsic research motivation and facilitates research progress. This finding supplements the two-dimensional challenge-hindrance stress framework with individual longitudinal evidence derived from intervention research.

From self-psychology, the self-structure has intrinsic tension and cohesion to buffer external stress and sustain psychological resilience. Long-term thesis work drains cognitive resources via heavy workload, outcome concerns and repeated revisions, gradually damaging self-structure integrity and inducing persistent research anxiety. To prevent mental collapse, students push themselves to keep researching, which further locks them into anxious states. The brief strategic withdrawal suspends energy-intensive hands-on research activities (literature review, thesis writing) to ease structural depletion and acute psychological strain. Though the relief is temporary and thesis completion remains pending, structured periodic rest yields lasting mental benefits. Aligned with Darwin’s rhythm of concentrated work paired with scheduled recovery, this intervention avoids excessive pressure buildup that would otherwise trigger psychological breakdown or self-structure fragmentation. Cycles of stress reduction and self-repair may cultivate context-specific academic resilience. Still, this single-case study cannot confirm a definite causal link between strategic withdrawal and resilience growth; relevant inferences about resilience cultivation remain preliminary.

This study is an exploratory, context-rich qualitative single-case report adopting an N = 1 exploratory single-case design without a control group. Confounding variables including inherent personality traits, thesis progress and unanticipated daily stressors cannot be ruled out to eliminate their interference with intervention effects. All conclusions are context-specific to this individual case only, and caution must be exercised when generalizing the findings to postgraduates across diverse disciplines, grades and research stages. In addition, only a two-week follow-up was arranged after the intervention, resulting in a relatively short observation window. Combined with RCI statistical results, long-term stable therapeutic effects of this intervention cannot be verified. Future research can expand the sample size, conduct randomized controlled experiments, and plan multi-stage long-term follow-up nodes in advance. In addition, external variables such as supervisor evaluations and institutional environments could be incorporated, and experimental outcomes could be assessed alongside physiological indicators (e.g., cortisol levels), thereby constructing a more comprehensive model of withdrawal effects.

## Conclusion

Based on the paradoxical intervention theory of Brief Strategic Family Therapy, this study developed a five-day brief strategic withdrawal intervention program to relieve research stress of master’s students in the middle stage of thesis writing. Mixed analysis combining three-wave scale measurements and participant diaries revealed that the intervention could alleviate research anxiety and reduce hindrance research stress, while no statistically reliable changes were observed in challenge research stress. This study provides a feasible practical reference for the adjustment of research stress among postgraduates.

## Data Availability

The original contributions presented in the study are included in the article/Supplementary Material. Further inquiries can be directed to the corresponding author.
